# Plateletworks^®^ as a Point-of-Care Test for ASA Non-Sensitivity

**DOI:** 10.3390/jpm11080813

**Published:** 2021-08-20

**Authors:** Hamzah Khan, Shubha Jain, Reid C. Gallant, Muzammil H. Syed, Abdelrahman Zamzam, Mohammed Al-Omran, Margaret L. Rand, Heyu Ni, Rawand Abdin, Mohammad Qadura

**Affiliations:** 1 Division of Vascular Surgery, St. Michael’s Hospital, Toronto, ON M4B 1B3, Canada; hamzah.khan@mail.utoronto.ca (H.K.); jains@ucalgary.ca (S.J.); muzammil.syed@mail.utoronto.ca (M.H.S.); abdelrahman.zamzam@unityhealth.to (A.Z.); mohammed.al-omran@unityhealth.to (M.A.-O.); 2 Keenan Research Centre for Biomedical Science, Li Ka Shing Knowledge Institute of St. Michael’s Hospital, Toronto, ON M4B 1B3, Canada; reid.gallant@mail.utoronto.ca (R.C.G.); Heyu.Ni@unityhealth.to (H.N.); 3 Department of Surgery, University of Toronto, Toronto, ON M4B 1B3, Canada; 4 Department of Laboratory Medicine & Pathobiology, University of Toronto, Toronto, ON M4B 1B3, Canada; margaret.rand@sickkids.ca; 5 Departments of Biochemistry and Pediatrics, University of Toronto, Toronto, ON M4B 1B3, Canada; 6 Translational Medicine, Research Institute, Division of Haematology/Oncology, The Hospital for Sick Children, Toronto, ON M4B 1B3, Canada; 7 Department of Medicine, McMaster University, Hamilton, ON L8N 3Z5, Canada; rawand.abdin@medportal.ca

**Keywords:** aspirin, resistance, non-sensitivity, antiplatelet, atherosclerosis, light transmission aggregometry, Plateletworks^®^

## Abstract

Aspirin (ASA) therapy is proven to be effective in preventing adverse cardiovascular events; however, up to 30% of patients are non-sensitive to their prescribed ASA dosage. In this pilot study, we demonstrated, for the first time, how ASA non-sensitivity can be diagnosed using Plateletworks^®^, a point-of-care platelet function test. Patients prescribed 81 mg of ASA were recruited in a series of two successive phases—a discovery phase and a validation phase. In the discovery phase, a total of 60 patients were recruited to establish a cut-off point (COP) for ASA non-sensitivity using Plateletworks^®^. Each sample was simultaneously cross-referenced with a light transmission aggregometer (LTA). Our findings demonstrated that >52% maximal platelet aggregation using Plateletworks^®^ had a sensitivity, specificity, and likelihood ratio of 80%, 70%, and 2.67, respectively, in predicting ASA non-sensitivity. This COP was validated in a secondary cohort of 40 patients prescribed 81 mg of ASA using Plateletworks^®^ and LTA. Our data demonstrated that our established COP had a 91% sensitivity and 69% specificity in identifying ASA non-sensitivity using Plateletworks^®^. In summary, Plateletworks^®^ is a point-of-care platelet function test that can appropriately diagnose ASA non-sensitive patients with a sensitivity exceeding 80%.

## 1. Introduction

Low-dose Aspirin (ASA), at 81 mg per day, is commonly prescribed for the secondary prevention of atherosclerotic/thrombotic adverse events such as myocardial infarction, cerebrovascular accidents, chronic limb threatening ischemia, and death [1,2,3,4]. It serves as first-line antiplatelet therapy among patients with coronary artery disease, carotid artery stenosis, and peripheral arterial disease [2,3,4,5]. The Antithrombotic Trialists Collaboration demonstrated that ASA is effective in reducing adverse cardiovascular events by approximately 20% [6]. However, an alarming 20–30% of patients with atherosclerotic disease suffer from ASA non-sensitivity, where ASA fails to prevent adverse cardiovascular events [7,8,9,10,11,12]. 

Plateletworks^®^ is a point-of-care platelet function test that measures platelet aggregation in response to specific platelet activators [13]. In this point-of-care test, citrated-whole blood is incubated in pre-prepared tubes containing different platelet agonists, specifically arachidonic acid (AA), adenosine 5’-diphosphate (ADP), and collagen. A control ethylenediaminetetraacetic acid (EDTA) tube establishes a baseline platelet count to which all other platelet counts are compared. After platelet aggregation is induced with a particular agonist, whole-blood hematology analyzers are used to determine the platelet count in each tube and calculate the percent maximal platelet aggregation. 

Plateletworks^®^ has a number of appealing strengths and features. First, it is an efficient test that can be completed within 10 min and that can simultaneously provide a complete blood count (CBC). Second, minimal training is required to conduct a platelet function test with Plateletworks^®^. Third, previous studies have utilized it in a variety of settings, fortifying its legitimacy and appeal [13,14,15,16]. With these strengths, Plateletworks^®^ is a potential point-of-care test that is easy, quick, and can readily be utilized in a variety of clinical settings. However, a cut-off point (COP) for ASA non-sensitivity using Plateletworks^®^ needs to be established before it becomes common practice in these settings. Therefore, the aim of this pilot study was to determine and validate the optimal COP to detect ASA non-sensitivity using Plateletworks^®^ in patients with established cardiovascular disease. 

## 2. Materials and Methods

### 2.1. Ethics Approval

This study was performed in accordance with the Declaration of Helsinki and was approved by the Unity Health Toronto Research Ethics Board at St Michael’s Hospital in Toronto, ON, Canada. Informed consent was obtained from each participant before enrolment in the study.

### 2.2. Patient Selection

A total of 100 patients were investigated in two successive phases: a discovery phase (*n* = 60) and a validation phase (*n* = 40). Patients with established cardiovascular disease presenting between September of 2019 and September of 2020 were recruited from St Michael’s Hospital’s outpatient vascular surgery clinic. Patients were eligible for inclusion if they were taking low-dose ASA (81 mg per day) for at least 14 days prior to recruitment. Patients that met any of the following criteria were excluded: (1) prior history of bleeding disorder, gastrointestinal bleeding, hemorrhagic stroke, thrombocytopenia, anemia, or leukopenia; (2) use of oral anticoagulants or antiplatelet medication other than ASA; (3) consumption of alcohol and/or another NSAID within the past 24 h and 3 days, respectively; (4) patients who are pregnant or nursing; (5) patients younger than 18 years of age. A negative control group, composed of patients with established atherosclerotic disease who had not yet been prescribed ASA or patients with venous disease not taking ASA, was also recruited. 

### 2.3. Baseline Measurements

A complete medical history and physical exam were performed on each patient. Medical history included details of any previous coronary artery disease, hyperlipidemia, hypertension, renal disease, congestive heart failure, diabetes, history of stroke or transient ischemic attacks, and smoking status. Baseline variables were defined as previously described [17]. Patients with a glycosylated hemoglobin A1c ≥ 6.5% or using anti-diabetic medication were classified as having diabetes mellitus (DM). Patients on anti-hyperlipidemic medication or having a total cholesterol > 5.2 mmol/L or triglyceride >1.7 mmol/L were classified as having hyperlipidemia. Patients using antihypertensive medication or with a systolic blood pressure ≥ 130 mmHg or a diastolic pressure ≥ 80 mm Hg were classified as hypertensive. Renal disease was defined as an estimated glomerular filtration rate of less than 60 mL/min/1.73 m^2^ [17].

### 2.4. Specimen Collection

Whole blood samples were drawn from the antecubital vein using a 21-gauge needle into 3.2% sodium citrate tubes to investigate ASA sensitivity immediately after collection. 

### 2.5. Gold Standard Light Transmission Aggregometry-Aspirin Sensitivity Testing

Light transmission aggregometry (LTA) is the gold-standard test for ASA non-sensitivity testing. To establish a COP for ASA non-sensitivity with Plateletworks^®^, each patient’s platelets were simultaneously tested with both Plateletworks^®^ and LTA using arachidonic acid (AA) activation to determine their ASA non-sensitivity status. LTA was conducted according to previously published protocols [11,18]. In short, platelet-rich plasma (PRP) was prepared by the centrifugation of citrated blood at 300× *g* for 7 min at room temperature, with deceleration set to 0. For PRP collection, 2 mm was cut off from the pipette tip to prevent the shear activation of platelets. Platelet-poor plasma (PPP) was prepared by centrifugation at 1200× *g* for 10 min at room temperature. PRP and PPP were transferred to fresh tubes and kept at 37 °C until testing. Platelet counts in PRP were collected using a Mindray BC-3600 21-parameter hematology analyzer (Mindray, Shenzhen, China) and adjusted to 2–3 × 10^8^ platelets/mL using autologous PPP. Platelet aggregation was performed with the stir bar speed set to 1000 rpm at 37 °C using a computerized aggregometer (Chrono-Log Corp, Havertown, PA, USA), with activation initiated with 0.5 mg/mL AA (101297, Bio/Data Corporation, Horsham, PA, USA). Patients were considered ASA non-sensitive if they had a residual maximal platelet aggregation ≥20% after induction with AA, as per previous studies [19,20,21,22,23].

### 2.6. Plateletworks^®^ Testing

Plateletworks^®^ was conducted as per the manufacturer’s protocol [13]. A total of 1 mL of citrated-whole blood was aliquoted into each Plateletworks^®^ tube containing either EDTA, collagen, AA, or ADP. Each tube was inverted gently 20 times to ensure the adequate mixing of the agonists. Platelet counts were obtained by running each tube on a Mindray BC-3600 21-parameter hematology analyzer (Mindray, Shenzhen, China). First, a baseline single platelet count was obtained by running the EDTA tube on the hematology analyzer. Immediately following the baseline platelet count, the ADP Plateletworks^®^ tube was gently inverted 5 times and a single platelet count was obtained. The collagen tube was gently mixed 5 times every 1 min for 4 min, after which a single platelet count was obtained. At the same time, the AA tube was gently inverted 5 times every 10 s for 2 min. The AA tube was then left at room temperature for 8 min, after which a single platelet count was obtained. The percentage of platelet activation by each agonist was then recorded using the following formula:

$$\mathrm{Aggregation}\;\left(\%\right)\;=\;100\;\frac{\mathrm{Baseline}\;\mathrm{Platelet}\;\mathrm{Count}-\mathrm{Platelet}\;\mathrm{Count}\;\mathrm{After}\;\mathrm{Activation}}{\mathrm{Baseline}\;\mathrm{Platelet}\;\mathrm{Count}}$$

### 2.7. Statistical Analysis

Demographics and clinical characteristics were expressed as means and standard deviations, or frequencies with percentages. Normality for continuous variables was assessed using normality plots and the Shapiro–Wilk test. Normally distributed continuous variables were reported with means and standard deviations. Median and interquartile ranges (IQR) were calculated for non-normally distributed data. Categorical variables were reported as counts and percentages. Pearson’s correlation coefficient was calculated to compare the percent of maximal platelet aggregation by LTA and Plateletworks^®^, with *p* < 0.05 considered statistically significant. We calculated the diagnostic performance of Plateletworks^®^ in detecting ASA non-sensitivity (i.e., sensitivity, specificity, positive predictive value and negative predictive value, and likelihood ratios) to determine the cut-off point for ASA non-sensitivity. All hypothesis testing was carried out at the 5% (2-sided) significance level. Statistical analysis was conducted using the GraphPad Prism software, version 8.4.2.

### 2.8. Optimal Cut-Off Point Selection for Predicting ASA Non-Sensitivity

To measure the sensitivity and specificity of Plateletworks^®^ in detecting ASA sensitivity at different cut-off values, a conventional receiver-operating characteristic (ROC) curve was generated. We calculated the area under the curve (AUC) to ascertain the quality of Plateletworks^®^ as a point-of-care test for ASA sensitivity. From the ROC analysis, the first identified maximal platelet aggregation value with a corresponding sensitivity of >80% was selected as a cut-off point to ensure a low rate of false negatives for ASA non-sensitivity.

## 3. Results

### 3.1. ASA Non-Sensitivity Cut-Off Point Discovery

#### 3.1.1. Patient Characteristics

In the discovery phase, a total of 60 patients were recruited, comprising 20 control patients not taking ASA and 40 patients on 81 mg of ASA daily. The median age for the discovery cohort was 67 years and the majority were male patients (72%). Noticeably, 62% of the discovery phase cohort were smokers, 66% suffered from hyperlipidemia, and 60% had hypertension. No recruited patients had thrombocytopenia, anemia, leukopenia, or any other blood disorders. Comparing these characteristics showed that patients taking 81 mg of ASA had significantly higher cardiovascular risk factors, such as hypertension, hyperlipidemia, and diabetes, compared to the control group not taking 81 mg of ASA (Table 1).

#### 3.1.2. Plateletworks^®^ Analysis

**Figure 1:**
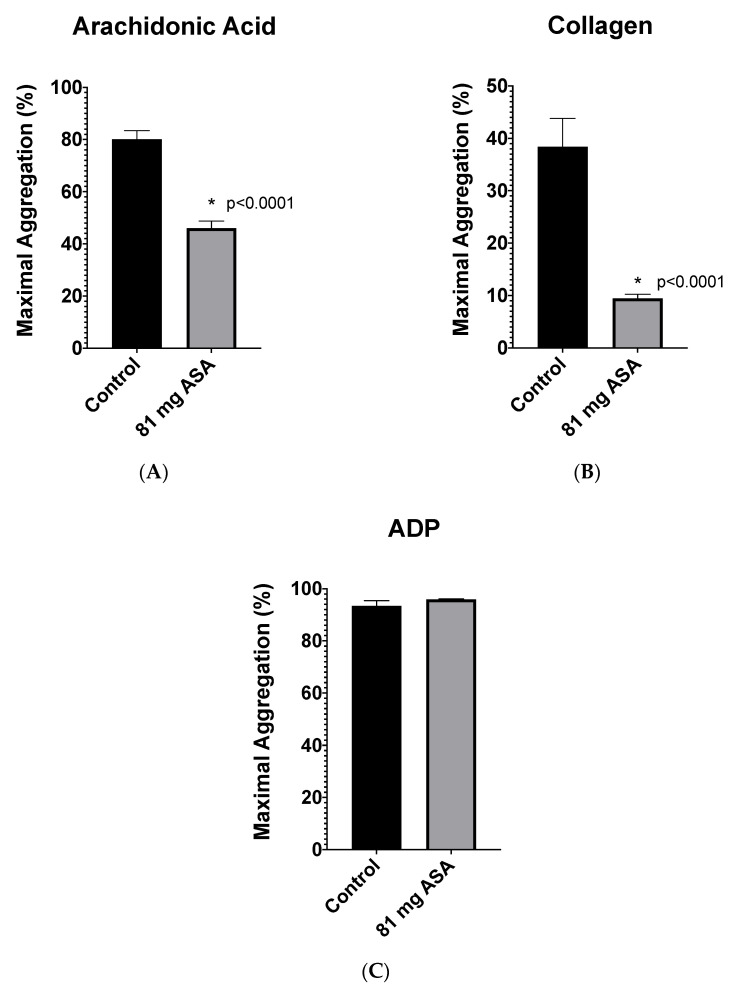
Plateletworks^®^ analysis between patients not on ASA (control, *n* = 20) and patients taking 81 mg aspirin (81 mg ASA, *n* = 40) using platelet activation agonists (**A**) arachidonic acid, (**B**) collagen, and (**C**) adenosine 5′-diphosphate (ADP). Error bars represent standard error of the mean. * represents significant difference between control and 81 mg ASA patients.

To investigate if Plateletworks^®^ could be utilized to distinguish patients on ASA from controls not taking ASA, Plateletworks^®^ analysis was conducted on all 60 patients recruited to the discovery phase. Relative to the controls, patients taking 81 mg of ASA had a significantly lower maximal platelet aggregation in response to activation with arachidonic acid (AA) and collagen (difference between means of 34 ± 4%, 95% CI: 26–43% and 29 ± 5%, 95% CI: 17–40%, respectively). We did not observe any significant difference in the maximal platelet aggregation between both groups post activation with ADP (Figure 1).

**Figure 2:**
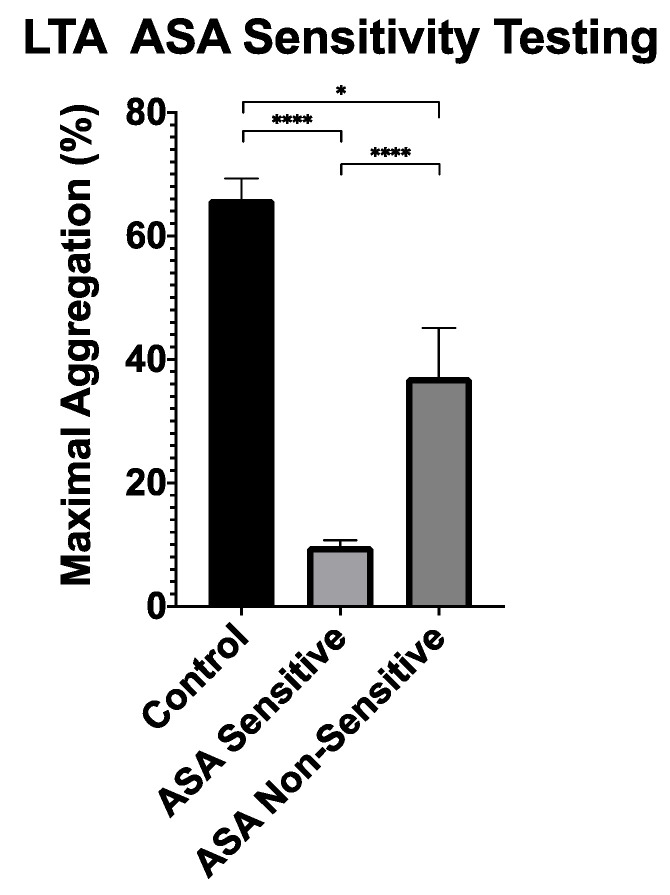
Light transmission aggregometry analysis between ASA sensitive patients (*n* = 30), ASA non-sensitive patients (*n* = 10), and controls not taking ASA (*n* = 20) with platelet activation using arachidonic acid. Error bars represent standard error of the mean. * represents *p* < 0.05, **** represents *p* < 0.0001.

To establish ASA sensitivity, the gold-standard LTA was utilized. All patients on 81 mg of ASA underwent LTA analysis with AA-induced platelet activation. LTA analysis demonstrated that 10 patients on ASA (25%) had a ≥20% maximal platelet aggregation, suggesting ASA non-sensitivity. As anticipated, all control patients who were not prescribed ASA had a maximal platelet aggregation ≥20% (Figure 2).

**Figure 3:**
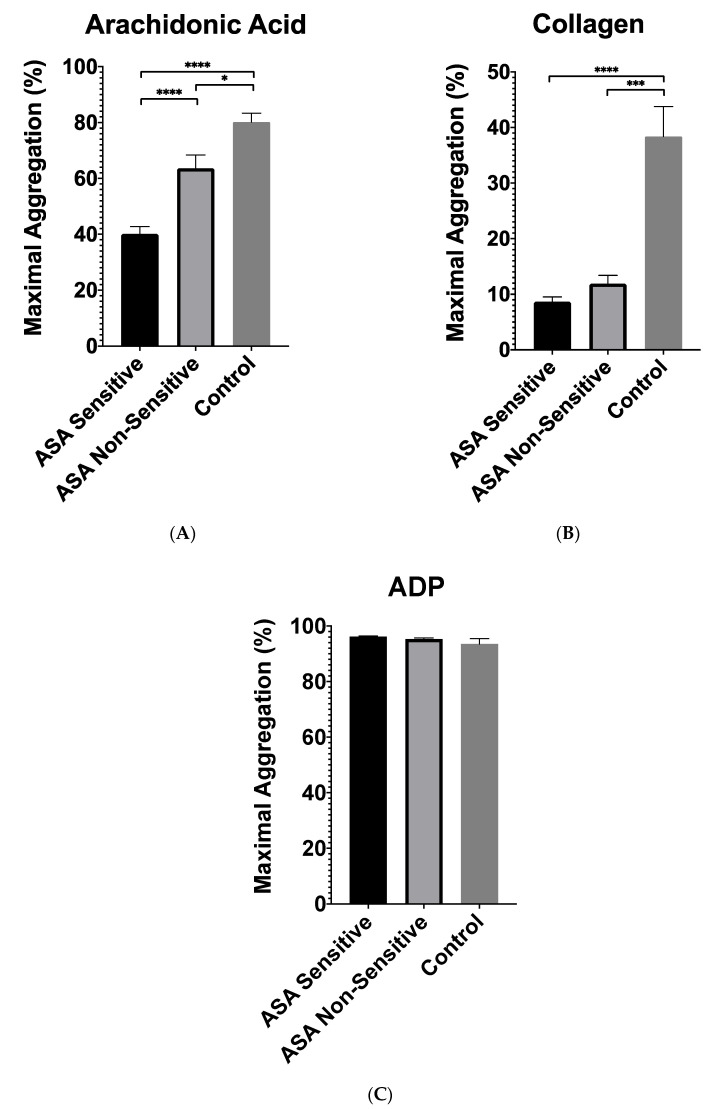
Plateletworks^®^ analysis between patients sensitive to 81 mg of ASA (*n* = 30), and ASA non-sensitive (*n* = 10) using platelet activation agonists (**A**) arachidonic acid, (**B**) collagen, and (**C**) adenosine 5’-diphosphate (ADP). Error bars represent standard error of the mean. Dotted line represents maximal aggregation in control patients not taking 81 mg of ASA. * represents *p* < 0.05; *** represents *p* < 0.001; and **** represents *p* < 0.0001.

Next, patients prescribed ASA were categorized into two groups based on their ASA sensitivity: patients who were sensitive to 81 mg of ASA (*n* = 30) and those who were non-sensitive to the prescribed 81 mg of ASA (*n* = 10). To assess Plateletworks^®^’ ability to detect a difference between ASA sensitive and non-sensitive patients, the Plateletworks^®^ results of both groups were compared (Figure 3). Relative to ASA sensitive patients, the ASA non-sensitive patients had a significantly higher response to AA, with a difference between means of 23 ± 5% (95% CI: 12–35%, *p* < 0.001). We did not observe any difference in response to collagen and ADP while comparing ASA non-sensitive to ASA sensitive patients (Figure 3).

**Figure 4:**
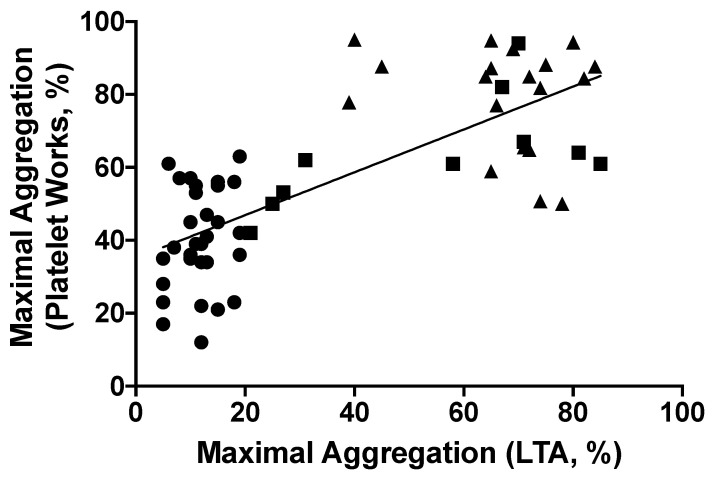
Pearson correlation between maximal platelet aggregation in response to Arachidonic acid using Plateletworks^®^ and light transmission aggregometry (*n* = 60). There is a good correlation between the two methods of platelet aggregation testing (r = 0.749, 95% CI: 0.6096 to 0.8432, *p* < 0.0001). Triangles represent patients not taking ASA (*n* = 20), circles represent ASA-sensitive patients (*n* = 30), and squares represent ASA non-sensitive patients (*n* = 10).

Next, we studied the correlation between maximal platelet aggregation in response to AA by LTA, with maximal platelet aggregation in response to AA by Plateletworks^®^ (Figure 4). Our analysis yielded a correlation coefficient of 0.75, suggesting a high correlation between Plateletworks^®^ and the gold-standard LTA (*p* < 0.0001) [24]. 

#### 3.1.3. Cut-Off Point Discovery

**Figure 5:**
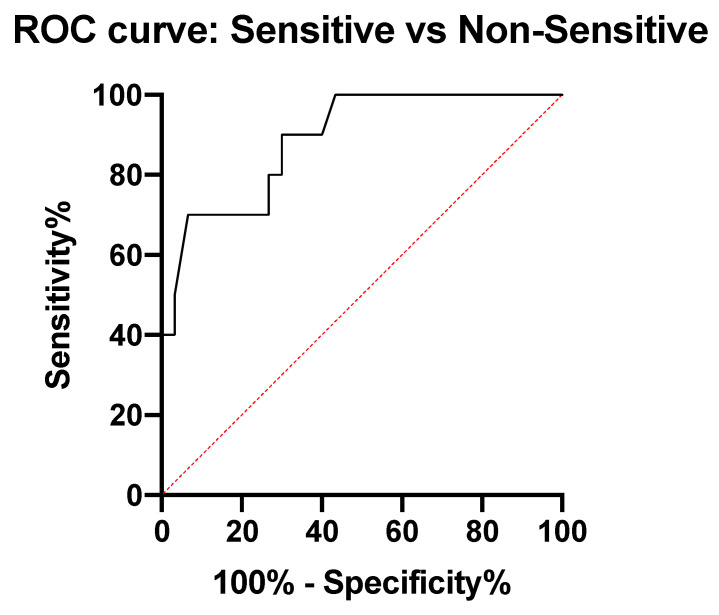
Receiver operating characteristics curve comparing ASA-sensitive patients (*n* = 30) to ASA non-sensitive patients (AUC = 0.89, 95%CI 0.78–0.99, *p* = 0.0003).

An ROC analysis was conducted that compared the percent maximal platelet aggregation in response to AA in Plateletworks^®^ between both ASA-sensitive and non-sensitive patients (Figure 4). Our data demonstrated that ASA non-sensitive patients had an area under the curve (AUC) of 0.89 (95% CI: 0.77 to 0.99, *p*-value = 0.0003) (Figure 5). Extrapolating the data from the ROC analysis, the first COP with a corresponding sensitivity of at least 80% was chosen. This value was identified to be >52% maximal platelet aggregation in response to AA, and hence was chosen as a diagnostic COP to distinguish ASA non-sensitive patients using Plateletworks^®^. This cut-off point had a sensitivity, specificity, and likelihood ratio of 80%, 70%, and 2.67, respectively.

### 3.2. ASA Non-Sensitivity Cut-Off Point Validation

#### 3.2.1. Patient Characteristics

A validation phase was conducted to confirm the COP of >52% maximal platelet aggregation using Plateletworks^®^ in predicting ASA non-sensitivity determined in the discovery phase. An additional 40 patients on 81 mg of ASA were recruited for the validation phase. Patients in the validation phase cohort had median age of 69 years, and primarily comprised males (63%). Notably, 75% of our study cohort were smokers, 85% suffered from hyperlipidemia, and 70% had hypertension (Table 2). No significant differences were noted between the patients on 81 mg of ASA in the discovery and validation cohorts in terms of all measured baseline demographics and clinical characteristics. 

#### 3.2.2. Cut-Off Point Validation

To investigate the diagnostic performance of the established COP, platelet aggregation post-AA stimulation was investigated using Plateletworks^®^. Each sample was simultaneously investigated and cross referenced with LTA. LTA analysis of the 40 patients showed that 11 patients (28%) were non-sensitive to 81 mg ASA. Our data demonstrated that the COP for Plateletworks^®^ of >52% maximal platelet aggregation post-AA stimulation has a sensitivity and specificity of 91% and 69%, respectively. This COP value was determined to have negative and positive predictive values of 95% and 53%, respectively, and a diagnostic accuracy of 75%.

## 4. Discussion

In this study, we demonstrated that Plateletworks^®^ was able to distinguish between patients who were non-sensitive to 81 mg ASA therapy from patients who are sensitive to 81 mg ASA. Using Plateletworks^®^, our results suggest a COP of >52% maximal platelet aggregation in response to AA has good sensitivity in identifying ASA non-sensitivity. We were also able to demonstrate that Plateletworks^®^ has a good correlation with LTA, the current gold standard.

ASA non-sensitivity has become a well-known and prevalent issue, with anywhere between 20 and 30% of patients at risk of further adverse cardiovascular events due to the failure of ASA to completely inhibit platelet aggregation. Previous studies have reported that 15% of patients with atherosclerotic vascular disease were non-sensitive to their prescribed 81 mg ASA therapy, and a further 32% of patients with cardiovascular risk factors were also non-sensitive to ASA [11,12]. There are two main mechanisms of ASA non-sensitivity: (1) pharmacokinetic variability in patients’ response to ASA—for example reduced absorption within the gastrointestinal tract, higher ASA metabolism, or increased platelet turnover; (2) pharmacodynamic differences such as genetic polymorphisms leading to structural changes in the COX enzyme [25]. A third reason why patients may be identified as non-sensitive is if patients are non-compliant with their ASA therapy. 

In this study, 21 of our 80 patients (26%) were non-sensitive to their therapy. This is a significant number, as these ASA non-sensitive patients have a ~6 fold increase odds of cardiovascular related death when compared to ASA sensitive patients [26]. Collectively, these data highlight the urgent need of a quick and easy, point-of-care test for ASA non-sensitivity that is not only available in hospitals, but also smaller clinical settings to allow for routine ASA non-sensitivity testing. 

LTA is the current gold standard for platelet function and ASA non-sensitivity testing [27,28,29]. It has a well-established COP for ASA non-sensitivity testing, specifically with patients with ≥20% maximal platelet aggregation in response to AA being considered ASA non-sensitive. Several studies have used this method for determining ASA non-sensitivity in both vascular and cardiac disease patients [19,20,21,22,23]. However, LTA can be time consuming, requires specialized equipment, and needs highly trained staff. LTA is not feasible for widespread use as a clinical ASA non-sensitivity test. There remains a need for a suitable quick, point-of-care test for ASA non-sensitivity, which can potentially be met by Plateletworks^®^. 

There are several point-of-care platelet function test currently available, however many have not been optimized to accurately detect ASA non-sensitivity. One such platelet function test is the Platelet Function Analyzer (PFA) 100/200. Citrated-whole blood is aspirated at high shear through a membrane coated with platelet agonists to mimic the in vivo environment of an injury. PFA-100/200, however, is an expensive test, has poor agreement with LTA, and the collagen/epinephrine cartridge (which is recommended for ASA non-sensitivity testing), and has shown to overestimate the prevalence of ASA non-sensitivity. One study demonstrated a sensitivity and specificity of 75% and 40%, respectively [20]. Another platelet function test that is becoming more commonly used in cardiac patients is the VerifyNow Aspirin test. In this assay, citrated whole blood is placed within cuvettes containing AA and microbeads coated with fibrinogen. Platelet aggregation is initiated and is recorded in Aspirin Reaction Units (ARU). A cut-off of >550 ARU has been recommended by the manufacturer as a cut-off for ASA non-sensitivity [30,31]. VerifyNow has been shown to be a good contender for ASA non-sensitivity testing in cardiac patients, with a high sensitivity and specificity demonstrated by several studies (100% and 96% respectively) [32,33,34]. However, some studies have shown a specificity as low as 35% [20]. There is a paucity of data regarding the use of VerifyNow within patients with vascular disease.

Plateletworks^®^ is a platelet function test that has potential to accurately detect antiplatelet non-sensitivity testing. The good correlation with LTA; its ability to be used on any hematology analyzer; its fast processing times (ASA non-sensitivity tests are run within 10 min); and the minimal training required for use are major strengths that suggest potential as an ideal point-of-care screening test for ASA non-sensitivity [13,15,35]. If more robustly proven, Plateletworks^®^ would enable physicians to conduct routine ASA non-sensitivity screening and inform physicians if further testing is required. Ultimately, by ensuring that patients are responding to their antiplatelet therapy, this test can help reduce the adverse cardiovascular events and intervention failures that occur due to ASA non-sensitivity. A potential COP for Plateletworks^®^ has also been suggested, with its feasibility demonstrated as an ASA non-sensitivity screening test. Our study demonstrates that Plateletworks^®^ has a comparable sensitivity and specificity to current point-of-care ASA non-sensitivity test frontrunners such as VerifyNow, and it shows a good correlation with the gold standard LTA.

This study has some limitations. First, a relatively small sample size was used; however, this study was designed to be a pilot that assesses the potential of Plateletworks^®^ as a point-of-care test for ASA non-sensitivity testing. In the future, larger studies need to be conducted to verify our COP for ASA non-sensitivity. Second, while patients were asked about ASA compliance, this was not formally assessed. Therefore, the non-compliance of patients nonetheless remains as a possibility. Despite this, Plateletworks^®^ still provides physicians with vital information about patients’ platelet activity, allowing physicians to further investigate the cause of their patients’ ASA non-sensitivity.

## 5. Conclusions

In conclusion, we demonstrated that Plateletworks^®^ may potentially be useful as a point-of-care screening test for ASA non-sensitivity using our validated COP in patients taking low-dose ASA (81–325 mg). While Plateletworks^®^ may not replace LTA as a gold-standard test for ASA sensitivity, Plateletworks^®^ can be utilized as a diagnostic screening tool or “pre-test” for ASA non-sensitivity, which could then be confirmed by LTA. It can also be helpful where LTA analysis is not available, such as in outpatient clinics in primary care or in resource-limited settings. Further clinical studies with larger sample sizes are required for validating this COP. With Plateletworks^®^, physicians will have easy access to point-of-care platelet function testing in a variety of clinical settings, allowing for fast and convenient ASA non-sensitivity testing. Additional research studies may use Plateletworks^®^ for investigating non-sensitivity to other antiplatelet agents that have also been found to be prevalent within the vascular population, such as clopidogrel [9]. The effective non-sensitivity testing of antiplatelet agents that can be performed with a point-of-care test such as Plateletworks^®^ will help to reduce the high risk of adverse cardiovascular events.

## Figures and Tables

**Table 1:** Baseline characteristics of patients recruited to the discovery phase.

Discovery Phase		
	Control Cohort (*n* = 20)	Patients on 81 mg ASA (*n* = 40)
		Mean (SD)
Age (yrs)	58 (19)	68 (9)
Platelet Count (10^8^/mL)	186 (62)	209 (70.47)
WBC (10^8^/mL)	6.2 (2.0)	6.8 (1.7)
HCT	0.380 (0.04)	0.384 (0.03)
		Frequency (%)
Sex (male)	15 (75)	28 (70) *
Hypertension	8 (40)	28 (70) *
Hyperlipidemia	8 (40)	32 (80) *
Diabetes	2 (10)	15 (37) *
Smoking	7 (35)	30 (75) *
CAD	0 (0)	12 (30) *
PAD	5 (13)	24 (60) *
		Medication (%)
Statin	9 (45)	33 (83) *
ACEi/ARB	6 (30)	21 (53) *
B-blockers	1 (5)	9 (23) *

Acetylsalicylic acid, ASA; white blood cells, WBC; hematocrit, HCT; angiotensin-converting enzyme inhibitors ACEi/Arb; coronary artery disease, CAD; peripheral arterial disease, PAD. Continuous variables are shown as mean (standard deviation). Categorical variables are shown as frequency (%). * represents significant difference (*p* < 0.05) between controls not taking ASA and patients on 81 mg of ASA.

**Table 2:** Baseline characteristics of patients recruited for the validation phase of the study.

Validation Phase	
Patient Demographics	Patients on 81 mg ASA (*n* = 40)
Mean (SD)	
Age (yrs)	68 (11)
Platelet Count (10^8^/mL)	185.3 (48)
WBC (10^8^/mL)	6.8 (1.9)
HCT	0.3832 (0.03)
Frequency (%)	
Sex (male)	24 (60)
Hypertension	27 (68)
Hyperlipidemia	33 (83)
Diabetes	20 (50)
Smoking	29 (73)
CAD	13 (33)
PAD	22 (55)
Medications (Frequency, %)	
Statin	34 (85)
ACEi/ARB	20 (50)
B-blockers	9 (23)

Acetylsalicylic acid, ASA; white blood cells, WBC; hematocrit, HCT; angiotensin-converting enzyme inhibitors/angiotensin receptor blocker ACEi/Arb; coronary artery disease, CAD; peripheral arterial disease, PAD. Continuous variables are shown as means (and standard deviations). Categorical variables are shown as frequencies (%).

## Data Availability

The data presented in this study are available on request from the corresponding author.
